# Enhancing empowerment in young adults during the COVID-19 era in Italy through the Photovoice technique

**DOI:** 10.1007/s12144-022-03635-5

**Published:** 2022-08-31

**Authors:** Nadia Rania, Ilaria Coppola, Marta Brucci, Laura Pinna

**Affiliations:** Department of Education Sciences, University of Genoa, Genoa, Italy

**Keywords:** Online Photovoice, Young adults, COVID-19, Empowerment, Italy

## Abstract

The spread of COVID-19 has led to increasingly stringent containment measures. After the first period of lockdown, there has been an easing of measures worldwide. However, this choice has helped bring about a second wave to be faced by many states. The present research was conducting during the period in which it was necessary for the population to find strategies for living with COVID-19. The proposed action research envisaged the use of Photovoice and, due to social distancing, was carried out online. A total of 250 young adults were involved with the aim of bringing out individual and community solutions for effective coexistence with COVID-19. The data, collected through a triangulation process, were analysed on the basis of grounded theory and the support of NVivo 12. The results highlight how online Photovoice is an effective tool for implementing individual and community empowerment and for identifying solutions to live with COVID-19.

## Introduction

The outbreak of the COVID-19 pandemic in 2020 resulted in severe restrictive measures, such as social distancing, aimed at countering the spread of the virus and limiting the health emergency. Countries have often been affected by natural or man-made disasters—i.e., disasters linked to a particular context—creating what is labelled in psychology as collective traumatic events (Hikichi et al., 2020; Rania et al., 2019). Hirschberger (2018) highlights how collective trauma is a psychological reaction that affects an entire society, a tragedy represented in the collective memory of the group, and which involves an ongoing reconstruction of the trauma in an attempt to find meaning in it. Therefore, also with regard to COVID-19 we can speak of collective trauma, as it is an event that, even if in different measures, has affected societies all over the world. Collective trauma can profoundly damage the social relationships that underlie the existence of a community (Frankenberg et al., 2012), and can lead to the arrest of institutional functions such as the economy or socio-political systems; these destabilizing effects can result in the impoverishment of social capital, negative psychological effects, hatred between factions and the deterioration of relations between people and the country, which tend to become increasingly rigid in their provisions (Price-Smith, 2009). According to Taylor (2019), pandemics can put a strain on states’ ability to cope with a health emergency for several reasons: the speed of spread of the virus, the lack of effective treatment, the difficulty in managing infected people or the infection of medical and health personnel. The economic costs of care and the lack of essential services can have severe consequences for families and entire social structures.

A problem encountered in the current emergency, which has made the picture of the situation even more complex, is the infodemic: that overabundance of information, which is not always accurate, that propagates through the interconnections of (mostly virtual) social media, making it is difficult for people to find reliable sources and reliable rules to follow to protect their health and that of their families and communities (WHO, 2020). In the information age in which we find ourselves, every phenomenon of global significance tends to spread to the point of becoming “viral” and, like viruses, affects our ideas and behaviours.

Working on individual and community empowerment can be a strategy to reinforce collective individual values and help people overcome this period of fear, anguish, and disorientation to which the COVID-19 health emergency has subjected populations across the world. Empowerment is an individual and collective growing process based on the increase of self-esteem, self-efficacy and self-determination and aimed to make individual able to consciously get his potential (Zimmerman, 2000). The use of coping strategies is useful to overcome difficulties for some reasons like being able to recognise the community context of change or focusing both on resources and deficits (Dickens & Groza, 2004). Moreover, empowerment strategies are effective because of the possibility they give to make individuals able to take action to change their situation (Gutiérrez, 1994) and to feel the sense of power (Cragg, 2018). Emphasizing self-determination through the practice of concrete goal-oriented strategies can produce social change (Bonilla et al., 2021) and at the individual level, can be useful in addressing health and well-being challenges due for example to the event. Pandemic that we have and are still facing today (Rania et al., 2019). Therefore, these are the reason why empowerment plays an important role in crisis such as the Covid-19 pandemic. Coronavirus disease has a lot of critical consequences, developing depression or anxiety symptoms (Glowacz & Schmits, 2020; Rania et al., 2021a) and the literature has shown that there has been a considerable increase in psychological stress among young people (Jackson & Lee Williams, 2022) with increased symptoms of anxiety and depression (Adams et al., 2022); furthermore, research carried out by Liu et al. (2022) highlighted that both during the first and the second pandemic 41% of young adults reported depressive symptoms and 47 anxiety. In this kind of situation, empowerment plays an important role and comes from the broader context. For this reason, the aim of this work is to highlight how, through an action research tool, the Photovoice, young adults were able to share strategies to overcome the difficulties related to COVID-19 and find possible solutions, developing individual empowerment and group.

### Photovoice to enhance empowerment, overcome trauma and find solutions

Participatory Action Research (PAR) is a methodology that involves an active role of the participants in the construction of knowledge (Merriam et al., 2001) following a process of “shared ownership of research projects, analysis of social problems on a community basis and a orientation towards community action “ (Kemmis & McTaggart, 2007, p. 273). A participatory approach is preferred when researchers want to know something about experiences that the participants lived, are affected by, or are involved in (Frisby et al., 2004). One of the aims of the Action-Research programs is to focus on problem-solving and to enhance a change effort and this is especially true for the PAR (Davison et al., 2021). In general, this kind of research is important in making the participants reflect on their lived situations and their daily lives with the aim of identifying possible solutions. This is particularly valuable and efficient in potentially dangerous situation, but it depends on creating trust and working together, something harder within the coronavirus disease. Because of these researchers are forced to innovate their instrument through the use of technologies and to make this kind of research more efficient even during COVID-19 pandemic (Hall et al., 2021; Rania et al., 2021b). The Photovoice method is a Participatory Action-Research (PAR) tool used to carry out community-based research projects. The Photovoice technique dates back approximately thirty years to Wang and Burris (1997). Photovoice is a participatory action research method based on arts-based methods (Coemans et al., 2015). According to Wang and Burris (1997), Photovoice has three main aims: (1) to bring out the strengths and weaknesses of one community through active listening and dialogue between participants; (2) to promote critical dialogue to the individual and community levels; and (3) to reach policy-makers and share with them the solutions identified to solve problematic or difficult situations in territories or communities. Although the photo is the fundamental point of the technique, in the first phase of Photovoice, each participant must, in fact, take the photos in their own context; however, the focus is not so much on the beauty of the photos but on what they want to convey. The images are used to initiate a dialogue on important issues, and the dialogue should translate into social change. The individual photographic activity is followed by a group discussion, called SHOWeD, with the analysis of participants’ pictures. Then, the participants prepared a final event in which they share with the stakeholders and wider community their empowerment and solutions to face the critical situations that they have discussed. However, according to Ronzi et al. (2016) and Migliorini & Rania (2017), the public exhibition of the results and the solutions identified by the participants do not always reach policy-makers. The SHOWeD phase consists of a guided group interview in which the moderator proposes the following questions to the participants while they look at all photos shared by participants: What do you *See* here? What’s truly *Happening* here? How does this relate to *Our* lives? *Why* does this problem or this benefit exist? How can we become *empowered* through our new understanding? What can we *Do* about this? The acronym SHOWeD is formed by the initials of the words in italics. Photovoice is an appropriate tool for investigating the complexity of communities and people’s relationships with their environment (Belon et al., 2014). Furthermore, in agreement with Sutton-Brown (2015), Photovoice extends the nature of photography from a visual art to a participatory social and political process, thus developing empowerment, according to the declaration of Zimmerman (2000), both on an individual level—when people reflect on the topic under investigation and engage in taking photos—and at the group level during the SHOWeD phase—when they build shared narratives and find solutions at the socio-political and community levels upon sharing with the wider community the path made through the final performance. Furthermore, Photovoice, as previously observed in relation to another trauma linked to the fall of the Morandi bridge in Italy (Rania et al., 2019), can be seen as a tool that allows participants to express deep emotions and feelings related to the tragedy, allowing them to work through the collective trauma and at the same time be an active part of the problem-solving process. Furthermore, Photovoice, through the discussion of one’s emotions and the search for potential solutions, can contribute to the development of social resilience, defined as the ability of social processes to respond to and recover from disasters (Aslam Saja et al., 2018). The strength of Photovoice, therefore, is not only in the images but also in the dialogic interpretations, which are realized during the discussion and solution phase; above all, the participants find the space to give voice to their own needs precisely with a view to empowerment by becoming more aware of their needs and the daily reality in which they live (Budig et al., 2018).

#### Aims

In this context, we present a research project of intervention on a community carried out during the period of the pandemic when the population tried to return to the normality of their routines and began to understand the need to have to live with Covid-19. The project aimed to stimulate participants to reflect and share strategies that they considered effective for living with COVID-19, with a view to individual and collective empowerment. Starting from a position of helplessness caused by having to face a pandemic crisis against which the single individual can feel helpless, through the sharing of photos and group discussions, the aim was to develop greater trust in the participants and in the potential of the group, as a tool activation of individual and group empowerment. A further objective was to find relevant and adequate solutions with a greater awareness of the problems faced and the possibility of changing the situation. With this project we have also set ourselves the goal of overcoming the collective traumatic event caused by the pandemic through the Photovoice technique that has emerged in the literature as a suitable tool for this purpose.

## Materials and methods

### Participants

A total of 250 (90% females) young adult volunteers from north-western Italy, with a mean age of 23 years (SD = 4.47, age range 19–40 years,), were involved in the research. Participants were recruited, on a voluntary basis, in various degree courses of the University of Genoa (Social Service, Psychology, Pedagogy, planning and educational research), to which the participants were enrolled. The teacher proposed a series of activities including the Photovoice and the students who attended the course were free to join. The only inclusion criterion was related to the fact of being enrolled in the course held by the teacher who managed the Photovoice activity. The wide participation was also due to the fact that the pandemic has upset everyone’s life and habits. Therefore, this activity represented an opportunity to reflect and share useful strategies to cope with the difficulties caused by COVID-19.

### Procedure

The activity was carried out through an online Photovoice technique, Participatory Activity Research (PAR), which was organized into four phases. The first phase in the plenary online approach was attended by 250 people divided into 4 large groups of approximately 60 people. Each meeting was reserved for a maximum of about 60 people to allow them an active participation. During this phase, the researcher presented the Photovoice methodology and the basics of the photographic technique. Moreover, the participants received information on the use of Photovoice images and gave written consent to voluntary participation. The participants were encouraged to think about the practical and emotional aspects of living with COVID-19. After the workshop, to encourage active participation in the subsequent phases, the participants were divided into smaller groups of about 4-9 members. Each group then followed the steps (phase 2-3 and 4). Phase 2 consisted of the participants taking photos of their daily experiences related to living with COVID-19 and selecting and commenting on two or three of their different photos taken individually. During phase 3, the participants, divided into groups, met on an online platform. Everyone presented their selected photos and explained the meanings of the photos and the feeling associated with taking the photo. The groups were then discussed following the “SHOWeD” method. The outcome of the discussion led each group to create a poster or a power-point presentation, which was presented in an online initiative with local stakeholders and shared on an online platform accessible to university students and their families (Phase 4). The study was conducted according to the ethical recommendations of the Declaration of Helsinki and the American Psychological Association (APA) standards for the treatment of volunteers. The data were collected in accordance with the Research Code of Ethics of the Italian Psychology Association.

### Data analysis

The researchers, through a process of triangulation of visual and textual data, analysed the photos, the respective comments and the transcripts of the SHOWED group discussions.

The researchers analysed and categorized the data, identifying the main common themes that emerged, using the software NVivo 12 (2019), which led to the creation of graphical representations based on grounded theory (Glaser & Strauss, 1967), a systematic and flexible methodology, which emphasizes data that is local and contextual and helps build models on empirical data. The theory is resumed and further developed by Charmaz (2014) subsequently takes up the theory and develops it further: the open coding of the data leads to the identification of the most significant codes, through an analytical process, which helps to identify recurring concepts and similarities in the data (Chun Tie et al., 2019). The main categorizations are identified. Starting from this theory, the authors analyzed the data, identifying themes and sub-themes, on the basis of the research question and the content provided by the participants on the theme proposed to them (Heydarian, 2016).

In analysing the data, the researchers assigned each participant an individual code (P1, P2, etc.) and a group code (SG1 = SHOWeD Group 1, SG2, etc.).

### Research team and relation with the research context

In the collection and analysis of these data it seems necessary to pay attention to the role played by the three researchers involved. The three members of the research group have different backgrounds and roles. The head researcher plays the role of associate professor within the Department of Education and guides the courses within which the activity from which the analyzed data was collected took place. The original idea of ​​the study stems from the desire of the head researcher and teacher to want to support her students in a very critical phase caused by the pandemic, helping them both to learn the contents of the course and to apply the tools covered by the course to develop individual empowerment and group. Since 2010, the researcher’s intent has been to share research-action techniques with students, applying an innovative teaching methodology that sees the active involvement of students. From this didactic experience the idea of ​​the research presented in the following paragraphs was born, which involved students of the socio-psychological area, whose participation took place on a voluntary basis. What most prompted the students to participate is due to the fact that this action-research activity represented a space within which one could virtually meet and reflect with one’s colleagues on the changes caused by the pandemic and on the psychological implications, while working to seek feasible solutions. In fact, the pandemic represented a trauma for the entire population, but in particular it had an impact on the psychophysical well-being of young adults, who had to adapt, limiting the moments of meeting and implementing new strategies to meet virtually and continue their lives. The other two authors, PhD students in Social Sciences at the time of data collection, were involved in the next phase of data analysis. With different educational backgrounds, one trained as a social worker and the other as a psychologist, they held the role of independent researchers, analyzing photos and transcripts of the stages of the discussion. Individually and starting from the grounded theory, the two authors codified the data and identified themes and sub-themes. Subsequently they met to discuss and define together the main problems that emerged and analyzed in this article. The authors were particularly involved in the action research process, as the situations reported and the solution strategies were centered on a theme that also involved them, like the whole community. However, the two independent researchers, having not taken part in the previous phases, were less involved in the reloading process and analyzed the data bringing their interpretation, then identifying the common themes. Where they could not find an agreement on the issues they confronted the researcher-teacher.

## Results

The findings, which emerged from the triangulation of visual and textual data, are presented and divided into actions to live with COVID-19 and solutions to address living with COVID through individual and community empowerment. Actions and solutions are divided into two subparagraphs. Regarding the actions and possible solutions various categories emerged which were represented through models of Nvivo 12, reported in the figures below. Furthermore, concerning actions for each theme and sub-theme some representative photos of that emerged are reported. The solutions are presented through three different models that represent the different levels of solution identified. Furthermore, the solutions are accompanied by the verbalizations of the participants as they emerged in the last phase of the SHOWeD from the comparison between them.

### Living with COVID-19: An opportunity to live

**Fig. 1:**
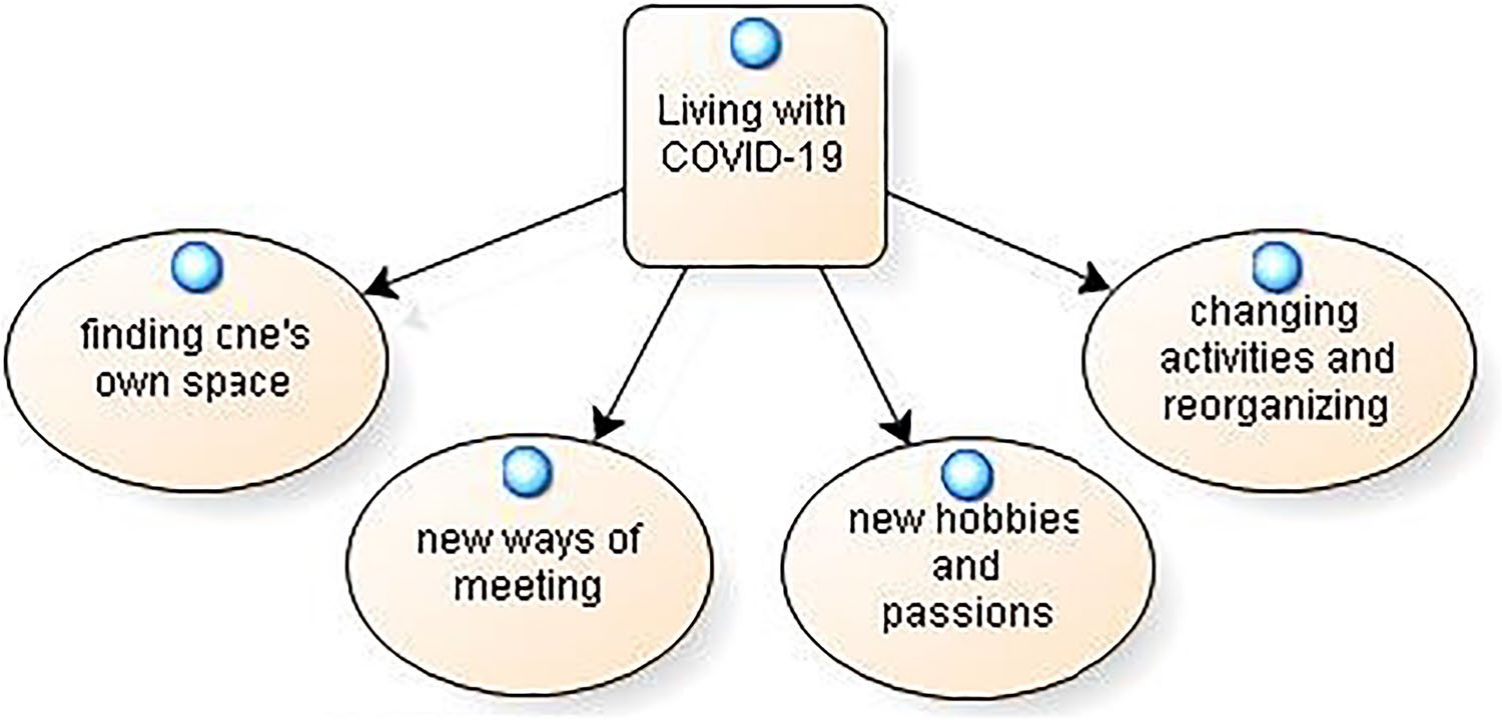
Living with COVID-19

From the analysis of the actions that the participants highlighted with respect to how people have adapted to everyday life with COVID-19, 4 categories were identified: changing activities and reorganizing, new ways of meeting, new hobbies and passions and finding one’s own space. The model is presented in Fig. 1.

#### Finding one’s own space

**Fig. 2:**
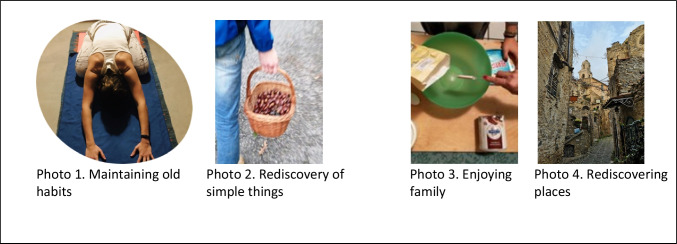
Finding one’s own space

Living with COVID has allowed people to maintain and/or find their own space through behaviours that have helped them to face changes related to the difficult circumstances. The following subcategories emerged: maintaining old habits, rediscovering simple things, enjoying family, and rediscovering places. Some participants pointed out that old habits were maintained during the lockdown. Photo 1, in Fig. 2, shows the continuation of sporting activity but at one’s home, which is necessary for one’s psychophysical well-being: “*Sport is health; it promotes an optimal lifestyle and protects the psycho-physical well-being of a person in every respect. Therefore, it is appropriate that we continue to do it even with the few tools we have available”* (P174). Others, on the other hand, underscored how living with COVID has led people to rediscover simple things, such as, for example, the activity shown in Photo 2 (Fig. 2), or being outdoors: “*rediscovery of simple things, committing time to being in fresh air”* (P49). While the lockdown forced people to stay at home, it nevertheless allowed them to enjoy their family. As reported in the verbalization to Photo 3 (Fig. 2), “*enjoy your family and appreciate at-home collaborations”* (P159). Finally, the pandemic and travel restrictions have favoured the rediscovery of localities nearby. Some participants highlighted through Photo 4 (Fig. 2) that “*the ban on moving from one region to another made it possible for many citizens to rediscover the beauty of places closer than one might think”* (P35).

#### New ways of meeting

COVID-19 and its implications have led people to find new ways of meeting. The following subcategories emerged from the triangulation of data from these new behaviours: maintaining social relationships online, playing with masks, deceiving distance, and attention at distances.

**Fig. 3:**
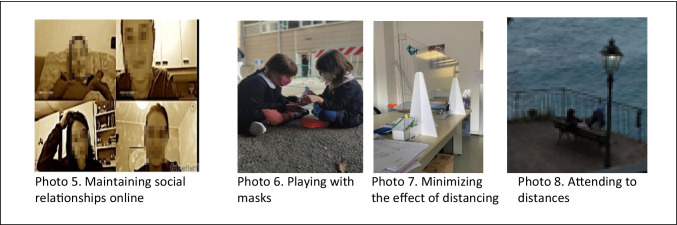
New ways of meeting

Social relationships have not been interrupted but rather have been maintained thanks to the help of social networks or dedicated platforms that have allowed a different daily life with respect to relationships. Photo 5, reported in Fig. 3, represents the “maintenance of social relationships online”. Starting from this photo, a participant wanted to underline how the pandemic has led to new ways of meeting safely: “*My friends and I all work with high-biological risk groups, and to protect our families, we decided to dedicate at least one time a week to seeing each other through online video calls. This is because the latest restriction of the DPCM (Prime Minister’s Decree) on COVID has taken away the possibility of seeing each other and talking over coffee. Surely, video calls can never replace a hug and a kiss, but it is the only temporary solution for us to be together. It is emotionally exhausting to be away from those you love, but knowing that I can hear them helps me to continue the week with serenity”* (P99). Even children have not interrupted their relationships, but during their play encounters, they have adapted to the new rules, such as wearing masks. In this vein, Photo 6 (Fig. 3) shows some children playing while respecting mask use. Some participants, starting from the photo discuss about this theme highlighting how in spite of new habits Children always find a way to play together: “*The two girls have fun all the same; now, the mask is a habit, school is a habit. The game is king, it does not matter anymore, the children do not care; they play and will continue to do so”* (P187). In many situations, it has been necessary to meet people in person, but everyday life has undergone transformations for reasons of safety and protection. As seen in Photo 7 (Fig. 3), transparent spacers have therefore been introduced in many contexts that require public interactions, thereby allowing the encounter with social distancing between people but minimizing the effect of distance. Starting from this type of photo, the participants shared how it is necessary to minimize the effects of the imposed distance in order to reduce the imposed separation: “*This emergency has led to other changes in our daily lives to increase safety. Staying away to protect yourself, trying to minimize the effect of this separation with transparent spacers, pretending that this distance does not truly exist, being able to talk to each other and looking at each other in the eyes. Being able to minimize the effect of the distance and separation that now must necessarily exist between people”* (P4). Living with COVID has allowed a redefinition of being together, which has come to include keeping distance and protecting oneself from the risk of contagion. In the verbalization of Photo 8 (Fig. 3), which represents a landscape, a participant wanted to photograph two people from behind, grandmother and granddaughter, who, while respecting the rules of distancing, chose to enjoy a few minutes together in the open air “*a grandmother and a grandson enjoy a few minutes in the air open together, always being careful to maintain their distance in accordance with established regulations and the risk of COVID contagion, given that there are two parties to protect. We try to live with the virus by maintaining the right relationships of closeness”* (P81).

#### New hobbies and passions

The new everyday life that has emerged following COVID has favoured people’s discovering or rediscovering hobbies or passions that have been realized in their individual or family context. From triangulation of the data, the following subcategories were highlighted: gardening, kneading pizza, painting, knitting, and playing trump.

**Fig. 4:**
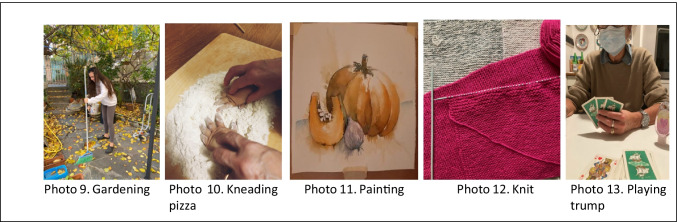
New hobbies and passions

As seen in Photo 9, reported in Fig. 4, gardening became one of the new hobbies highlighted by the participants and carried out during the lockdown, which allowed them to occupy their time at home in a different way, distracting themselves from the situation linked to COVID. Starting from this type of photo, the participants reflected together on how this type of hobby was initially a distraction and then became a passion: “*During the first lockdown, I started gardening in my small garden, watering the plants, planting seeds that had sprouted. Initially, this activity was a way to detach your mind from exams and lessons, but above all from the global pandemic that has touched us all, but later, it became a real passion”* (P123). Furthermore, staying at home has allowed family relationships to be redefined through hobbies that have favoured the realization of grandparent-grandchild and parents-child activities such as preparing pizza together (Photo 10, Fig. 4) and cultivating common hobbies, such as painting (Photo 11, Fig. 4), knitting (Photo 12, Fig. 4) or playing cards with the family (Photo 13, Fig. 4). Through photo 10, a participant wanted to share how the pandemic situation has allowed people to spend more time with their relatives “*a grandmother and a grandchild together in a home setting, preparing pizza dough. This single representation shows a positive aspect of how people have learned to live with COVID-19, which has led millions of Italians to spend almost all of their days at home. In particular, what has benefited in this case is the grandmother-grandchild relationship, which has been strengthening day by day”* (P49).

In Photo 11, a participant presents a picture of what she painted with the help of her mother and explains through verbalization how the pandemic situation prompted her to try: “*I had never painted before, but the current situation prompted me to try. My mother has been painting for a long time, and she helped me with that. With her help, I drew in pencil, and then I used the watercolour technique”* (P157).

Photo 12 and the related verbalization, on the other hand, shows knitting done by a participant: “*An activity that kept me company even during the first lockdown was knitting, a pastime that my mom had taught me a long time ago but that I had put aside. In this situation of “forced confinement“, I learned to appreciate this hobby, especially because it allowed me to spend many afternoons in the company of my mother, sitting on the sofa, with a cup of tea and good music in the background*” (P215).

As can be seen from photo 13 and from the verbalization of the participant, playing card games has also been an important activity allowing the maintenance of meaningful relationships and the resumption of normality. “*A Sunday tradition: the game of trump with my aunt, an elderly woman who lives alone and for whom I am her only niece, so we have always had a strong bond. During the first lockdown, I had not been able to visit her, and I was very sorry to know that she was alone in dealing with this situation. I tried to resume this tradition of ours and return to keep her company, no longer with the light-heartedness of the past but with many precautions: sanitizing gel and always double-masking, being very careful not to get too close to her and keeping the windows open so that the air can always circulate”* (P66).

#### Changing activities and reorganizing

**Fig. 5:**
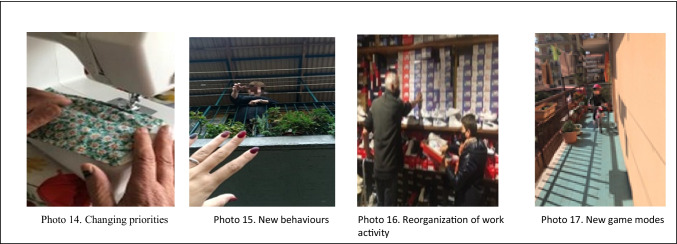
Changing activities and reorganizing

One of the most significant behaviours to have emerged from adapting to life with COVID-19 is the changing activities and reorganization of one’s life. From triangulation of the data relating to these behaviours, the following subcategories can be highlighted: changing priorities, new behaviours, reorganization of one’s work activity, and new modes of play. With respect to changing priorities, the pandemic has changed daily needs, and masks have become fundamental. In fact, through photo 14, reported in Fig. 5, the participants want to communicate how “*this situation has changed priorities: masks have become more important. This is why an 80-year-old lady went from sewing clothes to sewing masks”* (P10).

Furthermore, COVID-19 has introduced new behaviours to facilitate the maintenance of meaningful emotional relationships and to protect loved ones. Photo 15 (Fig. 5), in fact, chosen by the participants, shows how new behaviors have been adopted to protect loved ones “*the love for grandparents. In this period of COVID, we need to protect them like never before. Affective relationships are extremely important, which is why it is necessary to implement behaviours different from the usual ones. These gestures make us understand how we should never take anything for granted, because the big things are found in the smallest gestures”* (P150). The working environment was also reorganized according to the new rules relating to social distancing and the mandatory health and hygiene regulations after the reopening of commercial activities. Furthermore the participants underline how photo 16 (Fig. 5) “Reorganization of work activity” represents the way in which traders had to reorganize their activities following step by step the hygienic-sanitary indications necessary to deal with customers; in the photo they are wearing a mask, which has now become indispensable in everyday life “(P32). Even children have had to adapt to this new reality by finding play strategies in their own homes and not meeting with other children. Participants chose photo 17 (Fig. 5) to show a new way of playing and then discussed how the children also had to adapt their habits “*a little girl playing with her bicycle on the terrace. During a time when it was impossible to meet children from her peer group, she had to adapt to new ways of playing. We know the importance of children’s inclusion of play in their lives, which is useful for the development of intellectual and relational skills. Due to the spread of COVID-19, the daily lives of the little ones and their approach to play also changed. Little thought was given to children because, at first, they were considered almost immune to COVID-19, but it was their lives that came to be most disrupted as natural moments such as play required daily change”* (P230).

### Possible solutions: from individual empowerment to community empowerment

From the discussion group, thanks to the SHOWeD method, many solutions emerged that the participants were able to identify to deal with this emergency situation, thus overcoming the perceived and experienced trauma. From an analysis of these, it was possible to divide them into three levels: solutions at the *microsystem, mesosystem* and *macrosystem levels*. The most illustrative verbalizations are reported for each solution identified.

**Fig. 6:**
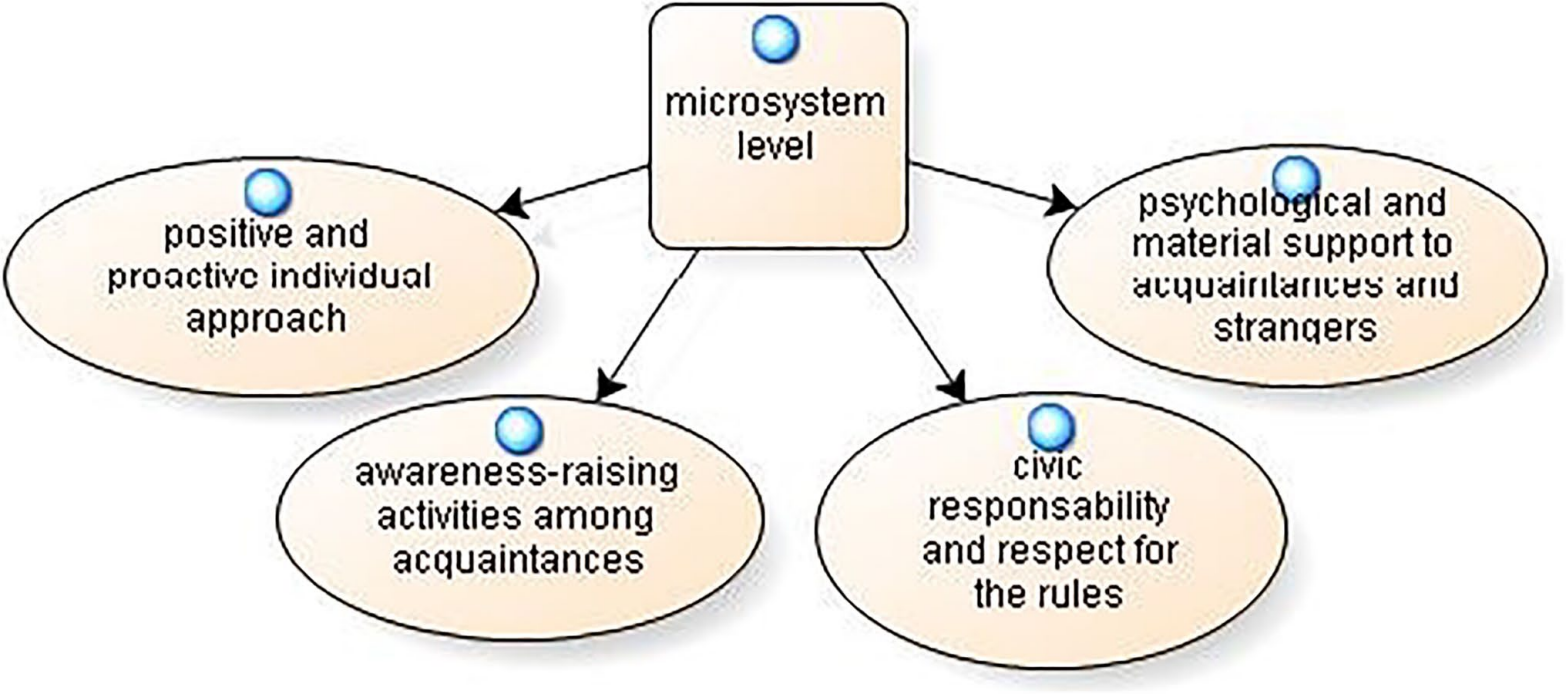
Possible solutions: microsystem level

The solutions found at the *microsystem level* can be grouped into 4 categories. The model is reported in Fig. 6.

For the “positive and proactive individual approach”, the participants underscore how it is necessary to “*remain attentive and focused on a single common goal, which is to stay healthy and to reorganize themselves in their own small ways, finding alternative solutions to what was currently denied to them*” (SG3). In comparison, “awareness-raising activities among acquaintances” are fundamental activities for the common good: “*Of course, perhaps the only thing we could do more is that, when we are faced with a person who does not respect these rules, to let him know this instead of, perhaps, not caring and saying ‘Oh, well nothing happens to a person here’; this is the only thing that comes to mind—so, yes, maybe creating greater awareness”* (SG1).

The proposed solution concerning “civic responsibility and respect for the rules” has the characteristic of *“taking care of others, like upholding one’s responsibility to and setting a good example for the world*” (SG20). “Psychological and material support to acquaintances and strangers” is seen as an essential resource: “*mutual support in general between acquaintances: for example, if your boyfriend or your friend has anxiety, mutual support—even at a distance, of course—can be provided with a phone call. However, there is also mutual support between strangers, those who have no relatives, and those who have nothing”* (SG15).

**Fig. 7:**
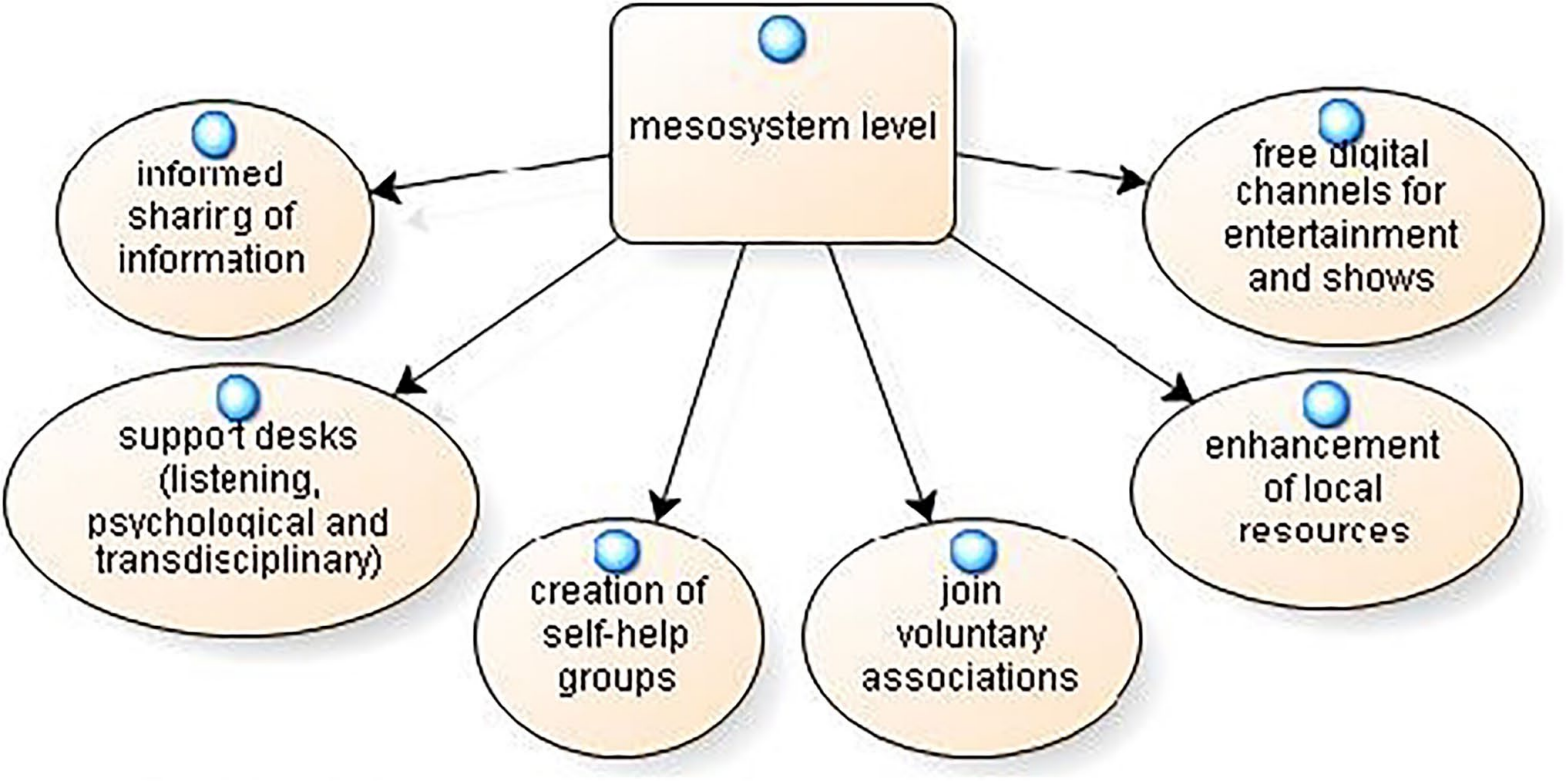
Possible solutions: mesosystem level

At the *mesosystem level*, participants identified 6 categories of solutions; the model is reported in Fig. 7.

Participants stressed the need for “informed sharing of information”: *“a Facebook group made up of. .. graduates or volunteers who simplify the information transmitted by the media”* (SG3). In comparison, the “support desks (listening, psychological and transdisciplinary)” solution emerges from the need “*to create psychological telephone support by institutions for people affected by COVID-19, [...] to set up a nucleus that works on the emergency, built through a transdisciplinary network of psychologists, educators, and doctors who can also offer telephone support”* (SG12). The “creation of self-help groups” still emerged as a solution “*maybe online for people in quarantine who are in the same situations”* (SG17). Other participants proposed to “join voluntary associations” *“who leave the grocery store or go to throw the garbage to people who cannot and do fundraising and food collections”* (SG25). The “enhancement of local resources” was seen as a solution to the economic crisis linked to COVID-19: “*The cinema—in particular, it’s being a community cinema—actually risked closing many times, and the neighbourhood community, when it found out, intervened by going to it”* (SG31). Finally, some participants identified the creation of “free digital channels for entertainment and shows” as a solution: “*Perhaps open more free channels that people can access to participate in artistic events, virtual art exhibitions, etc. where people can also engage in dialogue with each other”* (SG38).

**Fig. 8:**
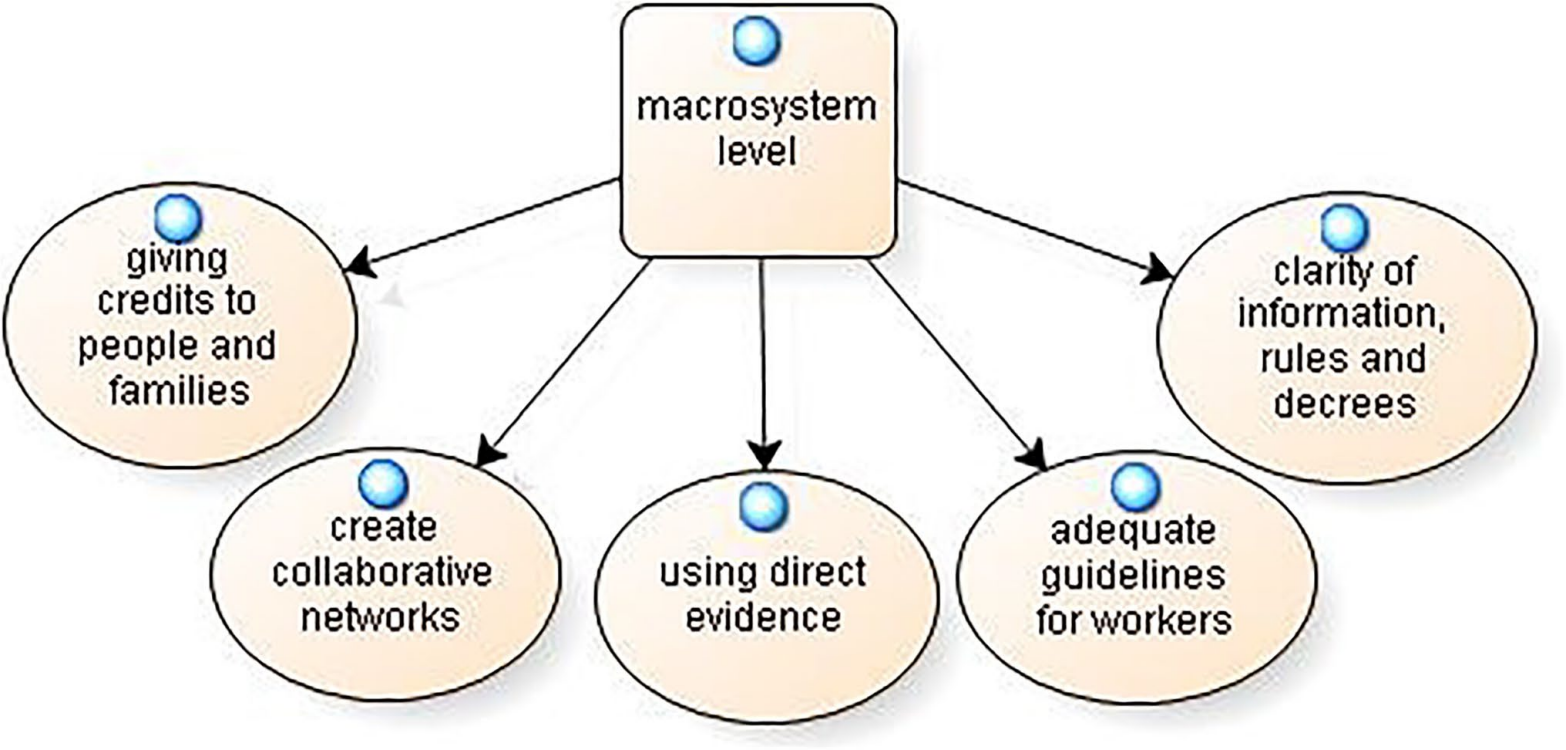
Possible solutions: macrosystem level

At the *macrosystem level*, 5 categories of solutions were identified, and are reported in the model in Fig. 8.

Young adults have proposed the solution of “giving credits to people and families”, for example, a “*psychological credits for free or low-priced interventions to support people in need, or a credit for internet access and the purchase of digital devices (to stay in touch with elderly and distant people); or even more aid for families who are in serious difficulty due to the lack of work”* (SG19).

Others expressed the need to “create collaborative networks”: **“***For example, pharmaceutical companies and various laboratories should work together to find a solution instead of waging war on each other over who wins the contract”* (SG28).

The solution to raise awareness among the population arises from the idea of “using direct evidence”: “*There is much awareness; however, we should focus more on raising awareness even among young people and not merely among the elderly, perhaps by showing them concrete situations of people who may have been ill or telling their testimonies; this is, in my opinion, the first step”* (SG22). Still others called for “adequate guidelines for workers”: “*guaranteeing hiring of unemployed people by employers who had suspended them due to the COVID-19 situation”* (SG12). Finally, the “clarity of information, rules and decrees” was also seen as a necessary solution for the well-being of the entire population: “*greater clarity in the presentation of the various decrees and resolutions by the entities, but above all greater consistency in the plan for implementing the rules at central and local levels. Better conveyance of news and, therefore, a greater selection of what is useful versus junk information in order to make the message less equivocal at the general level of the population”* (SG13).

## Discussion

This study highlights how, from triangulations between the visual and textual data, the individual participants took action to underscore how the changes in living with COVID led them to continue their lives using different strategies. Furthermore, participation in a Photovoice activity allowed them to find solutions on different levels by developing a process of individual and community empowerment. Through the realization of the Photovoice, the participants made use of group resilience, which affected the emotional, behavioral and psychological aspects determined by the traumatic situation experienced (Hikichi et al., 2020). Reflecting together and taking action to find solutions as required by the Photovoice technique, allows you to develop empowerment and overcome traumatic situations as emerged in the literature (Rania et al., 2019).

The solutions at an individual level are linked to the behaviours highlighted by the participants while living with COVID. In particular, the activities mainly carried out or raised as examples by and the testimony of the participants highlighted the need for active change to be able to live with COVID-19. This need has touched various aspects of life: changes in priorities, for example, has brought to light what the new needs are. The literature (Stallard et al., 2021) has described how an event such as COVID-19, experienced as trauma, can lead to recognizing one’s vulnerabilities and re-evaluating one’s personal priorities. Behaviours have also been influenced and adapted in the name of protecting loved ones.

An interesting node concerns work activity: many workers, in fact, had to quickly reorganize themselves according to the new rules. However, living with COVID-19 has also led to experimenting with new play strategies, an essential activity for children, despite the pandemic. Novins et al. (2021) have highlighted the effects of the pandemic on children, the need for flexibility and improvement in the quality of children’s lives via their families and their resilience in living with COVID.

The COVID-19 pandemic and the restrictive measures aimed at containing the spread of the virus have not led to a halt in social relations, but to a new way of living them. As highlighted by Rania & Coppola (2022) the population has shown more or less favourable attitudes to the social distancing imposed; in the present research many participants had resorted to new technologies or had maintained higher levels of protection to maintain their emotional, social and working ties. The literature (Riva et al., 2020) has noted that the need to share one’s values raises the need for a sense of connection, also through online exchanges, which is fundamental in containing the pandemic. It emerged that it was important during life with COVID to maintain one’s hobbies and passions or to practice new ones even in the company of significant others, especially family members: gardening, cooking, and painting were some examples of how people got active and had experimented with new activities for their physical and mental well-being. In this regard, a previous research highlighted how the family, as an instrument that generates trust in the other, in its adherence to the rules of protection and containment of viruses, represents the most functional group of mutual protection from contagion (Rania et al., 2022).

If there were many strategies and behaviours identified for one’s individual well-being, just as many solutions emerged at the individual level that also had repercussions at the social and community level. Starting from the need to adopt an individual and proactive approach, the participants also highlighted how individually, to promote the well-being of the community, some actions could be carried out as awareness-raising activities to support the rules in force that were seen as necessary for the common good and to maintain mutual support among acquaintances and especially towards those who were alone. This strategy arose from a perspective of civic responsibility and respect for the rules. Additionally, in the literature, it has emerged how neighbourhood social support through voluntary groups and committees during COVID-19 has contributed broadly to mitigate psychological distress through its anti-stress effect (Chen et al., 2021).

Beyond identifying those individual-level actions that a single person could implement for the common good, the participants also identified main group-level solutions. On the one hand, some concerned a conscious sharing of information; on the other, they focused on creating self-help groups, for example, or participating in volunteer activities, which could support persons finding themselves in difficult situations due to the pandemic. Qian and Hanser (2020) have also found that services offered by neighbourhood committees and volunteer groups can support residents via mitigating the negative impact of the epidemic on residents’ mental health. Furthermore, the need to contribute to enhancing the economic and noneconomic resources of the area was considered important; in fact, living with COVID-19 has had a significant impact on the global economy. The limitations put in place to curb COVID-19 have nevertheless created serious damage to the economy of various nations, with a negative impact on the Gross Domestic Product (GDP; NT Pramathesh et al., 2020). Moreover, numerous participants pointed out that in the absence of outdoor social activities, streaming channels could instead allow people to participate in virtual artistic or entertainment events wherein individuals could share the experience with each other. Indeed, some authors have found that companionship and emotional support online during COVID-19 have played an important role in alleviating negative emotions (Yao et al., 2021), especially the mothers seem to have used these strategies of solidarity network of care as shared literary / musical / cinematographic advice or created online content to entertain those who were at home (Rania & Coppola, 2022). From triangulation of the data, it emerged that the participants also identified solutions at the macrosystem level, focusing on the need for intervention by institutions to support and raise awareness and to involve the community in relevant pandemic-related considerations. On the one hand, the need to financially support people in difficulty and to guarantee respect and protection for workers was underlined; as was, in fact, found by Bierman et al. (2021), the economic hardships experienced during the pandemic have probably had causal consequences on the mental health of the population. On the other hand, more from a critical point of view, the idea was advanced that greater collaboration between institutions was needed to find solutions. Cheng et al. (2021) also found that live broadcasting through social media platforms proved to be an effective interactive tool for healthcare professionals to provide accurate information related to COVID, thus preventing the spread of incorrect information and alleviating anxiety in the population.

Finally, raising awareness of the population through direct testimonies and greater clarity of the regulations in force were seen as necessary solutions for the well-being of the population. Finally, all the solutions identified by participants, at the different levels, have the objective of contrasting the individual and collective malaise that the literature has shown to be a constant in the various age groups, without significant gender differences (Authors, 2021; Ahmed et al., 2020), but, in particular, among young adults (Rania et al., 2021a; Lee et al., 2020).

## Limitation and strengths of the study

Although this research has led to interesting results, there are some limitations: the first limit is related to the fact that the majority of participants are female; however the focus of this work was not centered on gender differences, but on how sharing solutions to overcome the trauma and difficulties caused by COVID-19 allowed young adults to develop empowerment. Another limitation is due to the educational background of the young adults who participated in the research, which may have influenced and facilitated the search and sharing of solutions and the development of resilience and empowerment. While staying aware of the limitations of our study, we can highlight that using Photovoice and the use of visual art in the form of photos, the participants faced this difficult, emotionally stressful moment by finding solutions (Hass-Cohen et al., 2018). Active participation in the choice of solutions and in planning and design can allow citizens the opportunity to take control of their own lives. The solutions identified by the participants were an attempt to reconnect with a sense of security and empowerment of one’s life, with both individual and community recovery process. The results found can be read within the restrictive measures adopted by the Italian government, which led the participants to identify effective solutions at different levels, micro, meso and macro, to live with COVID-19 and the measures imposed by governance, activating a process of individual and community empowerment.

## Conclusion

The consequences of trauma are usually associated with negative outcomes; however, in the literature (Northfield & Johnston, 2022), it has emerged that such experiences can also lead to positive changes. The use of the photovoice, as a participatory action research technique, allowed young adults to confront each other and share what they were experiencing, identifying possible solutions to live with COVID-19. Therefore, the young adults learning and using the technique at the same time used their own resources and made them available to the group, thus activating a process of individual and group empowerment. Furthermore, participation in the research has allowed them to have a virtual space within which to discuss, reflect and confront each other which has allowed them to strengthen social ties despite the distance imposed to counter the spread of COVID-19. Furthermore, Badanta et al. (2021), in discussing the use of Photovoice during COVID, highlighted the development of a sense of responsibility towards the community (for example, toward healthcare workers) via coping mechanisms, such as good humour and entertainment, and offering support to manage stressful work situations.

The use of the Photovoice methodology creates a space for critical reflection through participatory approaches, which favours, in informal contexts, the development of group resilience by affecting emotional, behavioural and psychological aspects, thereby mitigating the emotional impact of uncertain and highly traumatic situations, such as living with COVID-19. Even during COVID-19, individual and group resilience emerged in dealing with this difficult situation (Novins et al., 2021), which led to the identification of strategies to mitigate the impact of the pandemic and to promote coping and well-being.

Worldwide, people’s economic and social lives have been severely impacted by the consequences of COVID-19. The results emerging from this research could stimulate stakeholders to undertake shared and collaboratively built solutions with the community with an eye toward individual and community empowerment.

## Data Availability

The data that support the findings of this study are available from the corresponding author, upon reasonable request.
